# Contribution of infection and vaccination to population-level seroprevalence through two COVID waves in Tamil Nadu, India

**DOI:** 10.1038/s41598-023-50338-3

**Published:** 2024-01-24

**Authors:** T. S. Selvavinayagam, Anavarathan Somasundaram, Jerard Maria Selvam, P. Sampath, V. Vijayalakshmi, C. Ajith Brabhu Kumar, Sudharshini Subramaniam, Parthipan Kumarasamy, S. Raju, R. Avudaiselvi, V. Prakash, N. Yogananth, Gurunathan Subramanian, A. Roshini, D. N. Dhiliban, Sofia Imad, Vaidehi Tandel, Rajeswari Parasa, Stuti Sachdeva, Sabareesh Ramachandran, Anup Malani

**Affiliations:** 1 Directorate of Public Health and Preventative Medicine, Government of Tamil Nadu, Chennai, Tamil Nadu India; 2 Institute of Community Medicine, Madras Medical College, Chennai, Tamil Nadu India; 3 University of California, San Diego, CA USA; 4 Artha Global, Mumbai, Maharashtra India; 5 University of Manchester, Manchester, UK; 6 World Bank, Washington D.C., USA; 7 University of Chicago, Chicago, IL USA

**Keywords:** Infectious diseases, Risk factors, Viral infection

## Abstract

This study employs repeated, large panels of serological surveys to document rapid and substantial waning of SARS-CoV-2 antibodies at the population level and to calculate the extent to which infection and vaccination separately contribute to seroprevalence estimates. Four rounds of serological surveys were conducted, spanning two COVID waves (October 2020 and April–May 2021), in Tamil Nadu (population 72 million) state in India. Each round included representative populations in each district of the state, totaling ≥ 20,000 persons per round. State-level seroprevalence was 31.5% in round 1 (October–November 2020), after India’s first COVID wave. Seroprevalence fell to 22.9% in round 2 (April 2021), a roughly one-third decline in 6 months, consistent with dramatic waning of SARS-Cov-2 antibodies from natural infection. Seroprevalence rose to 67.1% by round 3 (June–July 2021), with infections from the Delta-variant induced second COVID wave accounting for 74% of the increase. Seroprevalence rose to 93.1% by round 4 (December 2021–January 2022), with vaccinations accounting for 63% of the increase. Antibodies also appear to wane after vaccination. Seroprevalence in urban areas was higher than in rural areas, but the gap shrunk over time (35.7 v. 25.7% in round 1, 89.8% v. 91.4% in round 4) as the epidemic spread even in low-density rural areas.

## Introduction

**Figure 1:**
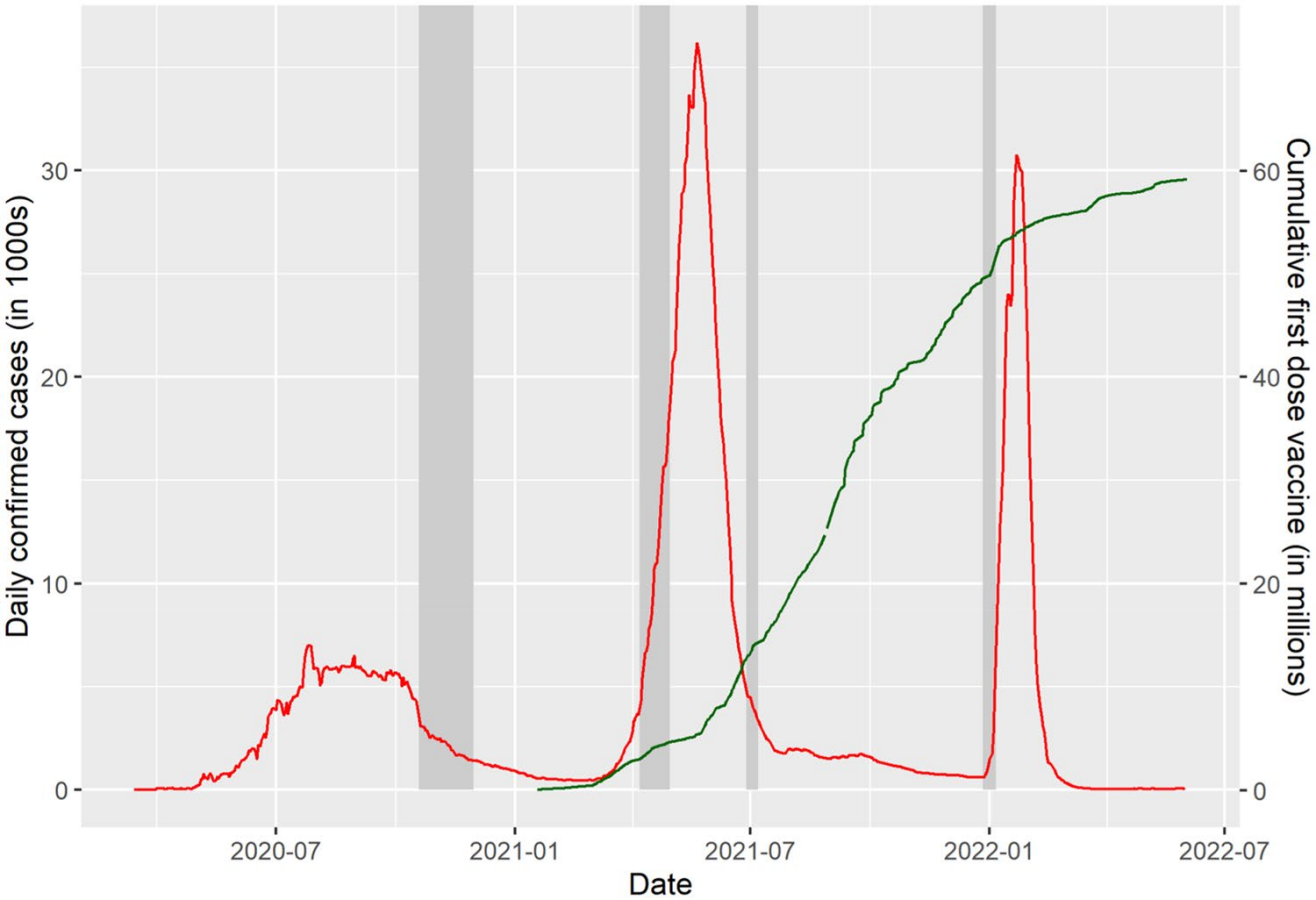
Daily new infections, new vaccinations (dose 1), and dates of serological survey rounds in Tamil Nadu. *Notes*: Figure plots daily confirmed cases (in 1000 s) in red on the left y-axis and cumulative number of first vaccine doses (in millions) on the right y-axis daily from February 2020 to May 2022. Dark grey intervals on x-axis indicate dates of each round of the serological survey in this study. Data source: www.covid19bharat.org.

Knowledge of population-level immunity is critical for understanding the epidemiology of SARS-CoV-2 (COVID-19) and formulating an infection control policy, including both non-pharmaceutical interventions and vaccination campaigns. To facilitate this policymaking, the state government of Tamil Nadu, conducted population-level serological surveys four times, in October–November 2020, April 2021, June–July 2021, and December 2021–January 2022 (shaded sections of Fig. 1). In each round, the survey was conducted on representative populations in every district of the state, except Chennai in round 2. Tamil Nadu is the 6th most populous state in India, with roughly 72 million persons^1^. India, including Tamil Nadu, experienced three COVID-19 waves that peaked in September 2020, May 2021, and February 2022^2^. Moreover, each state in India began a vaccination campaign in January 2021. Roughly half the population, 714 million persons, were vaccinated by February 1, 2022^3^. Thus, the state’s four serological surveys span the period between the end of the first wave and the start of the third wave and mid-point of the country’s first vaccination campaign.

This study reports seroprevalence estimates from these surveys over time, by district, by demographic groups, and by urban status. We employ these estimates to address three challenges of translating this surveillance into policy-relevant conclusions. First, not all changes to population-level immunity after a vaccination campaign starts are attributable to vaccination because the campaign takes time, during which the epidemic continues to infect the unvaccinated^4^. Moreover, spread of natural immunity can impact both the urgency and geographic focus of vaccine campaigns^4^. We address this problem by estimating the fraction of population immunity attributable to vaccination versus infection using a formula that compares changes in seroprevalence to changes in rates of infection and vaccination to across rounds of surveys.

Second, humoral immunity to SARS-CoV-2 from either infection or vaccination can be short-lived. Knowing how long it lasts is critical to determining the future value of non-pharmaceutical interventions and the urgency of both initial vaccination and boosters. There are many studies examining waning antibody levels in longitudinal samples, especially post-vaccination^5–14^. However, with few exceptions from Europe^15–17^, those studies do not example population representative samples^18–27^. Moreover, durability of humoral immunity may vary across the population^28^, so population-representative samples are critical for minimizing bias^29^. We estimate the extent to which antibodies decline following infection and vaccination by using data on changes in district-level seroprevalence across rounds and individual reports of the date of their own infection and vaccination, respectively.

Third, officially reported SARS-CoV-2 cases are typically not gathered from population-representative samples. Moreover, low testing rates may cause case counts to underestimate population-level immunity^30^. India reported 43 million COVID-19 cases and 524,000 COVID-19 deaths through May 31, 2022^2^. Tamil Nadu has reported roughly 3.4 million COVID-19 cases and 38,000 deaths, ranked 4th highest among Indian states through May 31, 2022^31^. Following similar exercises in other Indian states^32,33^, we compare the results of Tamil Nadu’s population-level serological surveys to these reported cases to measure the degree to which reported cases underestimate population immunity.

## Methods

The study was approved by the Directorate of Public Health and Preventive Medicine, Government of Tamil Nadu, and the Institutional Ethics Committee of Madras Medical College, Chennai, India. The study was performed in accordance with the guidelines of the Institutional Ethics Committee. In particular, only subjects who provided written informed consent were enrolled in the study, were surveyed, and asked for biosamples. The study was entirely funded by the Government of Tamil Nadu and the National Health Mission, Tamil Nadu.

### Outcomes

The primary endpoints are (1) the fraction of the population that would obtain positive results on CLIA (chemiluminescent immunoassay) antibody tests for COVID, i.e., seropositivity, at the district-level, and (2) the fraction of the population that have antibodies for COVID, i.e., seroprevalence, district level.

The secondary endpoints are (1) seroprevalence (a) by age and sex, (b) by urban status, and (c) at the state level; (2) the difference between population immunity estimated by serological survey and by reported cases; and (3) self-reported infection and vaccination.

### Survey timing, sample, and location

Data was gathered between 19 October–30 November 2020, 7–30 April 2021, 28 June to 7 July 2021, 27 December 2021–6 January 2022 in rounds 1, 2, 3 and 4, respectively. Individuals residing in Tamil Nadu and ages 18 years and older were eligible for rounds 1–3 of this study. In round 4, eligibility was expanded to ages 10 and older because the state government felt it safe enough to test for seropositivity among children. The exclusion criteria were refusal to consent and contraindication to venipuncture. In round 2, Chennai district was not surveyed because there was an outbreak that prevented sampling in that district.

### Sample size

Sample sizes for rounds 1–3 were calculated assuming a seropositivity of 0.5 throughout the state, to maximize power. For round 4, the positivity rate estimated from round 3 (0.662) was used. Calculations sought a confidence level of 0.95. Because clustered sampling would be done, a design effect of 1.5 was applied in rounds 1–3 and of 2 in round 4. The resulting sample size was multiplied by 37, the number of districts in Tamil Nadu as of October 2020, for rounds 1 and 3. In round 2, the multiple was 36 because Chennai was not sampled. In round 4, the multiple was 38, as one of the districts was split into two by round 4. This implied state-wide sample size targets were 26,651 in rounds 1 and 3, 25,931 in round 2, and 32,664 in round 4.

### Sampling strategy

The study selected participants in each district in five steps. *First,* districts were divided into rural and urban strata. District-wise sample-size targets were allocated to rural and urban strata in proportion to strata population. *Second,* rural and urban strata were divided into geographic clusters, defined as a village and street segments in rural and urban strata, respectively*. Third,* strata-wise sample-size targets were converted into cluster sample-size targets assuming 30 persons were sampled per cluster. *Fourth,* random sampling was used to select the targeted sample-size of clusters from each strata in each round. *Fifth,* up to 30 were sampled from each cluster using a random starting point, systematic sampling of households, and the Kish^34^ method to select one participant per household. (Additional details are in the Supplement).

### Data collection

Each participant was asked to complete a health questionnaire (including questions on prior infections and vaccination) and provide 5 ml venous blood collected in EDTA vacutainers. Serum was analyzed for IgG antibodies to the SARS-CoV-2 spike protein using either the iFlash-SARS-CoV-2 IgG (Shenzhen YHLO Biotech; sensitivity of 95.9% and specificity of 95.7% per manufacturer)^35^ or the Vitros anti-SARS-CoV-2 IgG CLIA kit (Ortho-Clinical Diagnostics; sensitivity of 90% and specificity of 100% per manufacturer)^36^. We obtained data on each reported COVID-19 case and death through May 2022 from the Government of Tamil Nadu and Covid19Bharat.org^31^ and on the number of tests done through January 2022 from the Government of Tamil Nadu.

### Statistical analysis

All statistics are calculated separately for each round unless otherwise indicated.

#### Seropositivity

The proportion of positive CLIA tests by district is obtained by estimating a logit regression of test result on district indicators and reporting the inverse logit of the coefficient for each district indicator. Observations are weighted by the inverse of sampling probability for their age and gender groups; the sampling probability here and below is based on population counts from the 2011 Indian Census. We reweight to match the 2011 Census because the Kish method ensures even (rather than representative) sampling by gender and age. Clustered standard errors are calculated at the cluster level.

#### Seroprevalence

Seroprevalence by district is estimated in two steps. *First*, we calculate the weighted proportion of positive tests at the district level. (We explain an exception for Chennai in round 1 and Virudhunagar in round 3 in the Supplement.) All samples in a district were tested using the same type of CLIA kit. We estimate a logit regression of test results on district indicators and take the inverse logit of the coefficient for each jurisdiction indicator. Observations are weighted by the inverse of sampling probability for their age and gender groups. Clustered standard errors are calculated at the cluster level. *Second*, for each jurisdiction, we predict seroprevalence using the Rogan-Gladen formula^37^, test parameters for the kit used in each jurisdiction, and regression estimates of seropositive proportion by jurisdiction.

State-level seroprevalence is obtained by aggregating the seroprevalence across districts weighted by 2011 Census data on the relative populations of districts.

Seroprevalence by demographic group is estimated in three steps. *First*, we calculate the proportion of positive tests at the jurisdiction-by-demographic group level in that round using logit regressions of test results on jurisdiction-by-demographic group indicators. Demographic groups indicators are sex x age for 6 age bins. Standard errors are clustered at the cluster level. *Second*, we predict district-by-demographic group level seroprevalence using the Rogan–Gladen formula. *Third*, we compute the weighted average of seroprevalence for each demographic-group across all districts. The weight for any given district is the share of the state-wide population in a demographic group that resides in that district. These weights are computed using data from the 2011 Indian census.

Seroprevalence by vaccine status in each of rounds 2–4 (when vaccines were available) is estimated in the same manner we calculate seroprevalence by demographic group in a round, except we replace demographic group by vaccine status.

The population was largely vaccinated with either of two vaccines. Covishield was developed by Oxford University, AstraZeneca and the Serum Institute of India. It is a viral vector vaccine that uses a modified version of a Chimpanzee adenovirus to deliver SARS-Cov-2 genetic material. Covaxin was developed by Bharat Biotech, the Indian Council of Medical Research, and the National Institute of Virology. It is an inactivated vaccine. Seroprevalence by specific vaccine in each of rounds 3–4 (when vaccine names were requested from respondents) is estimated in the same manner we calculate seroprevalence by demographic group in a round, except we replace demographic group by vaccine brand.

Seroprevalence by urban status is obtained in the same manner as seroprevalence by demographic group, with two changes. First, we use the urban status of a cluster in lieu of demographic status of an individual at each step. Second, observations in our regression are weighted by inverse of the sampling probability for their urban status.

The size of a population that was seropositive by the end of a round is obtained by multiplying our seroprevalence estimates for the population in that round by the size of that population (as reported in the 2011 Census).

#### Undercounting of infections

The degree of undercounting of infections in round 1 is estimated by dividing the estimated number of people that are seropositive in the Tamil Nadu population by the number of government-reported cases in that population as of 1 week before the median sampling date of that round (23 October 2020). We focus on round 1 because vaccinations started after round 1 and some seropositivity in rounds 2–4 is due to vaccination, not infections. The lag accounts for the delay, both between infection and seropositive status and between infection and prevalence testing. We calculate the Pearson’s correlation coefficient between undercounting rate and testing rate (tests per million as of median date of testing) by district.

#### Waning antibodies

We estimate the decline of antibodies after infection and in the absence of vaccination using district-level observations and a linear regression of district-level seropositivity in round 2 on district-level seropositivity in round 1. We focus on round 1 because no participants were vaccinated before round 1, meaning all seropositivity is due to infection. Observations are weighted in proportion to the population of each district in the 2011 Census. To address the possibility that decay is masked by new infections or vaccinations, we estimate a second specification that includes as controls a measure of the percent of population infected between round 1 and round 2 and the fraction of respondents who self-report vaccination. The measure of infection, which we call the “adjusted cases rate”, is the number of new confirmed cases per capita between rounds 1–2, adjusted by the infection undercount rate in round 1 (seroprevalence rate in round 1 divided by cases per capita until round 1).

We estimate the decline of antibodies following two doses of vaccination in two steps. *First,* we restrict the sample to individuals from round 4 who had been vaccinated with their second dose at least 20 days prior to bio sample collection. The 20-day delay is intended to omit the period of time during which antibodies are climbing post-vaccination. We do not consider individuals from round 2 because we do not have their date of vaccination and from round 3 because so few individuals were vaccinated by that date*. Second*, we estimate a linear regression with an indicator for whether a person was seropositive as the dependent variable and the number of years (i.e., number of days/365) since dose 2 as the independent variable. Observations are weighted to match age and gender proportions in the 2011 Census. To obtain plausibly causal estimates, we use age as an instrumental variable (IV) for the number of days since vaccination. The logic for this instrument is that Tamil Nadu prioritized individuals for vaccination based on their age, with older age persons given greater priority; to validate this instrument, we create a binscatter of days since vaccination on age among individuals with only 1 dose of vaccine and confirm that days since vaccination rises with age. The drawback of this instrument is that it is possible that antibody decay is directly a function of age^38^.

#### Attribution to infection or vaccination

We attribute the change in seropositivity from round t − 1 to round t to changes in the levels of infections and of vaccination using the formula:

1 $$1=\frac{({s}_{t}^{v}-{s}_{t-1}^{v}){p}_{t-1}}{{s}_{t}-{s}_{t-1}}+\frac{({s}_{t}^{nv}-{s}_{t-1}^{nv})\left(1-{p}_{t-1}\right)}{{s}_{t}-{s}_{t-1}}+\frac{({s}_{t}^{v}-{s}_{t}^{nv})({p}_{t}-{p}_{t-1})}{{s}_{t}-{s}_{t-1}}.$$

where s_t_ is the seropositivity rate in round t, p_t_ is the fraction of the sample vaccinated by round t, s^v^_t_ is the seropositivity rate among those vaccinated by round t, and s^nv^_t_ is the seropositivity rate among those not vaccinated by round t. The first term captures the share of the change in seropositivity (s_t_ − s_t-1_) attributable to changes in seropositivity rate among the previously vaccinated (s^v^_t_ − s^v^_t-1_). (This rate can change over time because antibodies levels may depend on the number of days since vaccination.) The second term captures the share attributable to infections among the previously unvaccinated (1 − p_t_). The third term captures the share attributable to changes in the vaccination rate. This captures both the effect of the increase in the vaccination rate (p_t_ − p_t-1_) and the change in seropositivity when one gets vaccinated (s^v^_t_ − s^nv^_t_). We calculate these components for the change in seropositivity from rounds 2–3 and from rounds 3–4.

Statistical tests comparing groups are performed using a two-sided Wald test with 95%. All statistical analyses were conducted with Microsoft Excel 365 (Microsoft, USA) and Stata 16 (StataCorp, USA). All plots were generated in R.

## Results

### Sample

In *round 1*, the study obtained results for 26,135 persons in 882 clusters (Table 1). The study could not sample 6 clusters and was unable to consent 324 persons in sampled clusters. One person aged 16 was incorrectly consented and dropped from the analysis. In *round 2*, the study obtained results for 21,966 persons in 746 clusters. (Chennai was not sampled.) The study could not sample 118 clusters and was unable to consent 388 persons in sampled clusters. Twenty-six persons of age < 18 were incorrectly consented and dropped from the analysis. In *round 3*, the study obtained results for 26,592 persons. The study could not consent 48 persons in sampled clusters. In *round 4*, the study obtained results for 32,244 persons. The study was unable to sample 13 clusters and could not consent 56 persons in sampled clusters. The final sample size per round was within the allowable 20% non-response rate.

**Table 1:** Demographics of sample, as compared to 2011 Census.

	Sample				
	Oct–Nov 2020	Apr 2021	Jun–Jul 2021	Dec 2021–Jan 2022	Census 2011
*Gender*					
Male	39%	42%	42%	42%	50%
Female	61%	58%	58%	58%	50%
*Age*					
10–17				6%	16%
18–29	23%	17%	19%	17%	26%
30–39	23%	21%	23%	20%	19%
40–49	20%	21%	22%	20%	16%
50–59	16%	18%	18%	18%	11%
60–69	11%	14%	12%	13%	8%
70 +	6%	8%	6%	7%	5%
*Obs*					
	26,135	21,966	26,592	32,244	

Census 2011 number for ages 18–29 includes only those ages 20–29.

Table 1 reports the demographic characteristics of the sample in each round. The sample has substantially more females and fewer persons aged 10–17 and 18–29 and more elderly persons than the general population.

### Seropositivity

**Figure 2:**
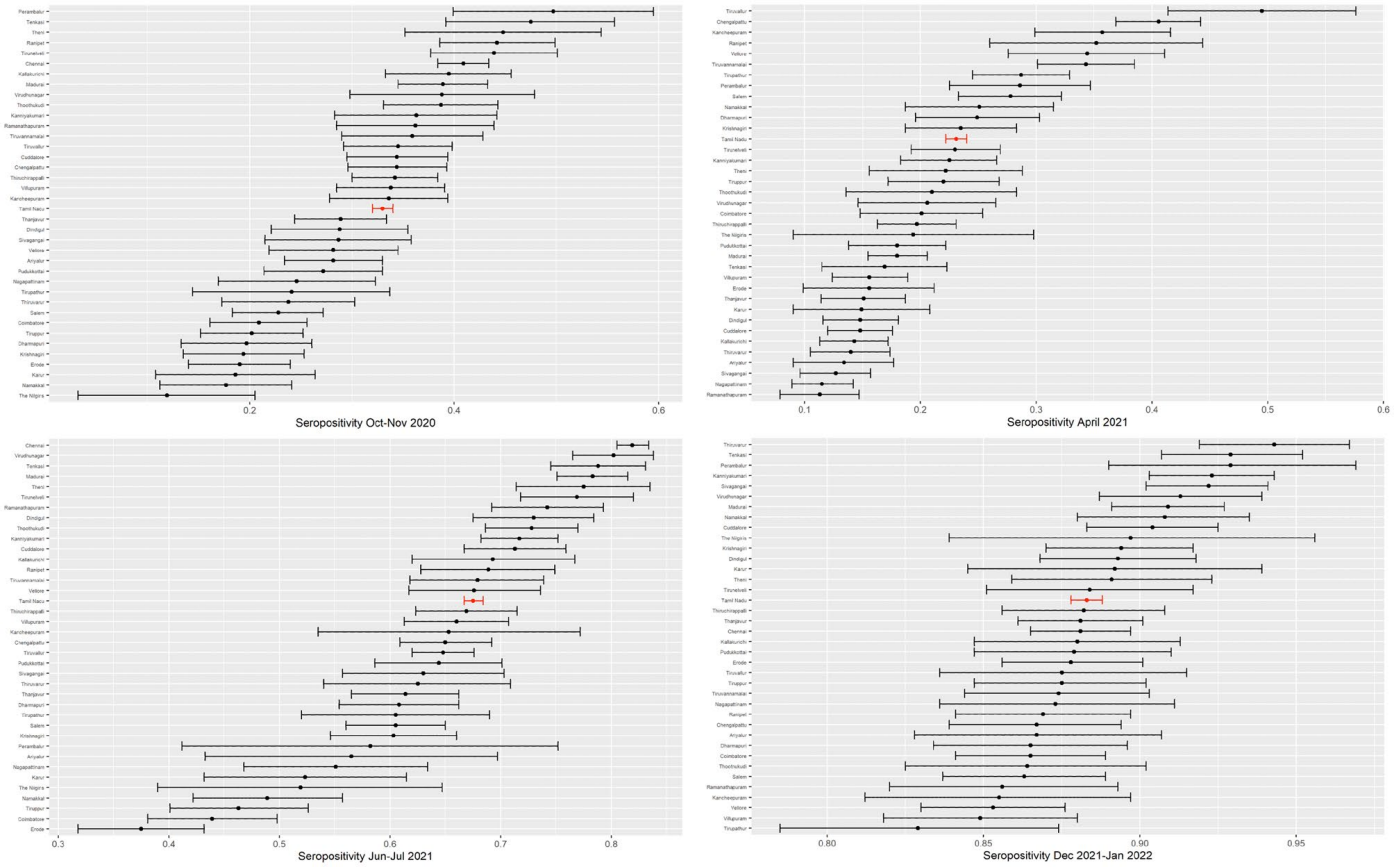
Proportion of positive CLIA tests by district. *Notes*: Each subfigure plots the mean (dot) and 95% confidence intervals (whiskers) of seropositivity on a CLIA test for antibodies to SARS-CoV-2 in each district (black) and in the state overall (red) in one round of the Tamil Nadu seroprevalence survey. Districts or state are indicated on the y-axis. Weighted proportion of sample is indicated on the x-axis, with weights indicated to make the sample representative of a district or state’s population, as appropriate. The date of each round is indicated in the subfigure titles.

State-level seropositivity was 33.0% (95% CI 32.0–34.0%), 23.1% (95% CI 22.2–24.0%), 67.5% (95% CI 66.7–68.4%), and 88.3% (CI 87.8–88.8%) in rounds 1, 2, 3 and 4, respectively (Fig. 2).

Seropositivity varied dramatically across districts in the first 3 rounds: from 11.9% (The Nilgris) to 49.7% (Perambalur) in round 1, 11.3% (Ramanathapuram) to 49.5% (Tiruvallur) in round 2, and 37.5% (Erode) to 81.9% (Chennai) in round 3 (Fig. 2). Seropositivity converged by round 4, ranging from 82.9% (Tirupathur) to 94.3% (Thiruvarur).

### Seroprevalence

State-level seroprevalence was 32.4% (95% CI 31.3–33.6%), 21.6% (95% CI 20.6–22.6%), 69.2% (95% CI 68.2–70.2%), and 90.6% (CI 90.1–91.1%) in rounds 1, 2, 3 and 4, respectively (Table 2). District-wise seroprevalence has a similar pattern to district-wise seropositivity (Fig. S1).

**Table 2:** Seroprevalence by type of region, sex, and age.

Variable		(1) October–November 2020			(2) April 2021			(3) June-July 2021			(4) Dec 2021-Jan 2022		
		Seropre- valence	CI lower bound	CI upper bound	Seropre- valence	CI lower bound	CI upper bound	Seropre- valence	CI lower bound	CI upper bound	Seropre- valence	CI lower bound	CI upper bound
State		32.4%	31.3%	33.6%	21.6%	20.6%	22.6%	69.2%	68.2%	70.2%	90.6%	90.1%	91.1%
Region	Rural	25.7%	24.3%	27.0%	NA	NA	NA	64.1%	62.9%	65.4%	89.8%	89.0%	90.6%
	Urban	35.7%	34.0%	37.4%	NA	NA	NA	74.8%	73.1%	76.6%	91.4%	90.7%	92.1%
Sex	Male	30.2%	28.8%	31.7%	21.3%	20.0%	22.6%	65.5%	64.2%	66.7%	88.5%	87.7%	89.3%
	Female	30.8%	29.5%	32.0%	22.0%	20.8%	23.1%	67.5%	66.2%	68.7%	92.7%	92.1%	93.3%
Age	10–17	NA	NA	NA	NA	NA	NA	NA	NA	NA	69.1%	66.4%	71.8%
	18–29	30.4%	28.7%	32.2%	19.5%	17.8%	21.1%	67.7%	66.1%	69.3%	92.2%	91.3%	93.2%
	30–39	30.7%	29.0%	32.5%	21.2%	19.6%	22.8%	67.0%	65.4%	68.6%	90.7%	89.7%	91.8%
	40–49	31.7%	30.0%	33.5%	22.1%	20.4%	23.9%	66.7%	65.0%	68.4%	91.8%	90.9%	92.7%
	50–59	32.2%	30.3%	34.1%	25.6%	23.8%	27.4%	66.6%	64.8%	68.5%	89.7%	88.7%	90.7%
	60–69	28.4%	26.2%	30.5%	22.8%	21.0%	24.6%	65.0%	62.8%	67.2%	87.8%	86.6%	89.1%
	70 +	26.5%	23.7%	29.2%	21.5%	19.0%	23.9%	59.6%	56.8%	62.3%	83.5%	81.6%	85.3%
Vaccine doses	0	30.5%	29.4%	31.6%	20.9%	19.9%	22.0%	62.3%	61.2%	63.3%	68.1%	65.8%	70.4%
	1	NA	NA	NA	25.7%	23.3%	28.1%	77.5%	75.6%	79.4%	87.4%	86.3%	88.4%
	2	NA	NA	NA	NA	NA	NA	85.9%	83.3%	88.4%	95.0%	94.5%	95.6%
Vaccine names	Covaxin	NA	NA	NA	NA	NA	NA	69.7%	65.3%	74.0%	83.7%	81.7%	85.7%
	Covishield	NA	NA	NA	NA	NA	NA	80.6%	78.9%	82.4%	94.1%	93.6%	94.6%

Table presents mean and 95% confidence interval bounds for seroprevalence for subgroups indicated in row titles for rounds indicated in column titles. Seroprevalence statistics are calculated from seropositivity on CLIA test for antibodies to SARS-CoV-2 spike protein using the Rogan-Gladden formula to correct for imperfect test accuracy. Samples are weighted to be representative of the state population in each subgroup.

Seroprevalence was significantly greater in urban areas than rural areas in rounds 1 (35.7% v. 25.7%, *p* < 0.001) and round 3 (74.8% v. 64.1%, *p* < 0.001) (Table 2).

Urban classification of clusters was not available for round 2. By round 4, however, the gap has largely closed (91.4% v. 89.8%, *p* < 0.001).

Seroprevalence is not substantially different across sexes (females v. males: 30.8% v. 30.2% in round 1; 22.0% v. 21.3% in round 2; 67.5% v. 65.5% in round 3; 92.7% v. 88.5%, round 4) (Table 2). While the round 4 difference is significant (*p* < 0.001), it is still a small gap.

Seroprevalence is highest among older working-age populations in rounds 1 to 2 and among younger populations in rounds 3–4. Seroprevalence is significantly higher among older working-age populations than the elderly in rounds 1–3 (age 50–59 v. age 70 + : 32.2% v. 26.5%, *p* = 0.002 in round 1; 25.6% v. 21.5%, *p* = 0.006 in round 2; 66.6% v. 59.6%, *p* < 0.001 in round 3). Seroprevalence among young adult populations is significantly greater than among the elderly in rounds 3–4 (18–29 v. 70+: 67.7% v. 59.6%, *p* < 0.001 in round 3; 92.2% v. 83.5%, *p* < 0.001 in round 4) (Table 2). However, seroprevalence among the children aged 10–17 is lowest of all in round 4 (69.1%, *p* < 0.001 v. each other age group).

Seroprevalence is significantly greater among vaccinated populations with any number of doses (25.7% v. 20.9%, *p* < 0.001; 80.0% v. 62.3%, *p* < 0.001; and 93.1% v. 68.1%, *p* < 0.001 in rounds 2, 3, and 4, respectively). Rounds 3 and 4 suggest that seroprevalence is increasing in number of doses taken (0 doses v. 1 dose: 62.3% v. 77.5% (*p* < 0.001) in round 3, and 68.1% v. 87.4% (*p* < 0.001) in round 4; 1 dose v. 2 doses: 77.5% v. 85.9% (*p* < 0.001) in round 1 and 87.4% v. 95.0% (*p* < 0.001) in round 4) (Table 2).

Seroprevalence varied by vaccine platform. Most sample members in rounds 3 and 4 received the Covishield vaccine (89.21 v. 10.59 Covaxin, with remainder getting other vaccines). Seroprevalence is greater among individuals who received the Covishield, as opposed to the Covaxin, vaccine (round 3 and 4 combined: 89.18% v. 80.08%, *p* < 0.001).

### Undercounting

The ratio of the number of infections implied by seroprevalence to confirmed cases is both high and varies widely across districts, from 10 to 148 in round 1 (Table S1). There is a significant negative correlation ($\rho$= − 0.58, *p* < 0.00) between COVID testing rate per thousand and the undercount rate in round 1 (Fig. S2).

### Waning antibodies

On average, district-wise seroprevalence rate in round 2 is 68.4% of the seroprevalence rate in round 1 in a district (Table 3), implying a 31.6% decline in seroprevalence, perhaps due to antibody waning. Across districts, the average adjusted cases rate is 9.35% between rounds 1 and 2 and on average 17.8% of sample members report being vaccinated with at least 1 dose by round 2. Adding (a) the district-level adjusted case rate to the regression to control for seropositivity due to new infection and (b) the self-reported vaccination rate to the regression to control for seropositivity due to vaccination yields a lower seroprevalence rate of 42.7% of round 1, implying a significantly larger 57.3% decline (*p* < 0.001) in seroprevalence after infection.

**Table 3:** Decay of antibodies following natural infection.

	(1)	(2)
	Dependent variable: seroprevalence in round 2	
Seroprevalence in round 1	0.684***	0.427**
	(0.0635)	(0.129)
Adjusted case count		1.204**
		(0.370)
Fraction vaccinated		− 0.143
With 1 dose by round 2		(0.161)
N	36	36

This table presents the results from regressing seroprevalence at the district level in round 2 against seroprevalence at the district level in round 1 and no constant. In column 2, the regression also includes the district-level adjusted case rate between round 1 and 2 to capture new COVID infections and the fraction vaccinated with 1 dose (self-reported) by round 2 to capture the new vaccination rate.﻿ *p < 0.05; **p < 0.01 ***p < 0.001.

The annual rate of antibody decay after vaccination among individuals given 2 vaccine doses by round 4 is 16.3 percentage points (p.p.) after 1 year (Table 4). The seropositivity rate falls to zero within a year with the use of age as an instrumental variable for time since dose 2 to obtain causal estimates.

**Table 4:** Decay of antibodies following vaccine dose.

	(1)	(2)
	Dependent variable: seropositivity in round 4	
Years since dose 2	− 0.163***	− 1.001***
	(0.0125)	(0.150)
Constant	0.960***	1.161***
	(0.00368)	(0.0359)
N	15,676	15,676
Total doses	2 doses	2 doses
Instruments	–	Age

This table presents results from regressing seropositivity against years since second dose of vaccine. In the second column, years since last dose is instrumented by age. *p < 0.05 **p < 0.01 ***p < 0.001.

### Attribution to infection or vaccination

Seropositivity increased by 42 p.p. between rounds 2, just before India’s second COVID wave, and round 3, after that wave (Table 5). Infections accounted for 74% of this increase. Increased seropositivity among those vaccinated by round 2 accounted for 23%, and new vaccinations accounted for just 2%. Seropositivity increased 23 p.p. from round 3–4, the period between India’s second and third wave. New vaccinations accounted for 65% of this increase. New infections and greater seropositivity among those vaccinated by round 3 accounted for 22% and 13% of this change.

**Table 5:** Attribution of seropositivity trends to infection or vaccination.

	Change in seropositivity	Antibody response given vaccination	Infection given no vaccination	New vaccinations
	$({s}_{t}-{s}_{t-1})$	$({s}_{t}^{v}-{s}_{t-1}^{v}){p}_{t-1}$	$\left({s}_{t}^{nv}-{s}_{t-1}^{nv}\right)\times \left(1-{p}_{t-1}\right)$	$\left({s}_{t}^{v}-{s}_{t}^{nv}\right)\times ({p}_{t}-{p}_{t-1})$
*Round 2*–*Round 3:*				
Percentage points	42	9.6	31.1	1.1
Percent of $({s}_{t}-{s}_{t-1})$	100%	23%	74%	3%
*Round 3*–*Round 4:*				
Fraction	23	3.0	5.2	15.1
Percent of $({s}_{t}-{s}_{t-1})$	100%	13%	22%	65%

Table present the total change in seropositivity (column 1) and the fraction of that change attributable to antibody response to vaccination (column 2), to antibody response to infection alone (column 3), and to new vaccinations (column 4). The attributions are based on the formula in Eq. (1). The changes are reported for the changes between rounds reported in row titles. The units of the change are reported in the row subtitles.

## Discussion

Our estimates of seroprevalence rates across the four rounds of our survey are in line with other cotemporaneous surveys conducted in Tamil Nadu. Serological surveys before our round 1 survey in October–November 2021 are all lower than estimated seroprevalence (32.4%) that round^39–41^. Our round 1 estimated seroprevalence is in the middle of the range of estimates from late 2020^39,42,43^. Likewise our round 3 estimate of 69.2% seroprevalence in June–July 2021 is similar to the one other study in mid-2021^42^. This is reassuring as our survey is the only state-wide survey conducted; all prior ones in Tamil Nadu focused on single districts. (No other surveys were conducted in Tamil Nadu in the time range of our round 2 and 4 surveys.)

Our estimates of seroprevalence depart from the literature in two respects. First, our survey is the first population-representative, repeated cross-sectional survey to show a decline in seroprevalence, consistent with antibody waning. Second, our survey suggests that Tamil Nadu had higher seroprevalence than India as a whole^44–48^.

Our seroprevalence estimates suggests that officially confirmed cases dramatically underestimate the number of infections before vaccination. Statewide seroprevalence in round 1 implies that at least 22.6 million persons in Tamil Nadu were infected after Tamil Nadu’s first wave (by 30 November 2020). This estimate of actual infections is roughly 35 times larger than the number of confirmed cases by round 1 (674,802 cases by 16 October 2020)^49^.

Our findings suggest that humoral immunity following infection declines rapidly. Seropositivity declined between 31.6 and 57.3% across districts over the roughly 6 months (170 days) between rounds 1 and 2 of Tamil Nadu’s survey, before the state’s vaccination campaign made substantial progress. An implication is that vaccination remained critical for humoral immunity despite India’s devastating Delta wave of infections.

Our study also finds declining seropositivity—implying declining antibody counts—after vaccination. However, we only observe individuals for at most 6 months after last dose. Therefore, one should not extrapolate from these data beyond one-half year. Moreover, participants with the greatest time since vaccination are also older, and older people may experience more rapid waning of antibodies^38^. Therefore, caution should be taken before extrapolating to younger populations.

Both infection and vaccination can contribute to seroprevalence. Between rounds 2–3, India experienced a second COVID-19 wave due to the Delta variant^50^. As a result, a majority of the increase in seropositivity from 23.1 to 67.5% was attributable to new infections. Between rounds 3–4, India ramped up its vaccination campaign and did not experience another COVID-19 wave. Therefore, most of the increase in seropositivity from 67.5 to 88.3% was attributable to vaccination. This suggests that, as of the last round of Tamil Nadu’s survey, the majority of humoral immunity was attributable to natural immunity from the Delta wave. The potential value of India’s vaccine campaign, while critical, was limited by the timing of the Delta wave. Our analysis also reveals that vaccination is associated with higher rates of humoral immunity than natural infection, and that this can explain a non-trivial portion of the growth in humoral immunity, even in round 3 (23%), when the vaccination rate was still low.

Our data suggest that additional vaccine doses may contribute to seroprevalence, but with effects diminishing in the number of doses. Moreover, the Covishield vaccine is associated with greater population seroprevalence than Covaxin, which is consistent with studies suggesting Covishield had greater vaccine efficacy^51,52^.

Our study has several limitations. First, because antibody concentrations in infected persons decline over time^53^, our estimate of seroprevalence in round 1 may underestimate the level of prior infection and perhaps natural immunity. Moreover, evidence that cellular memory persists beyond humoral immunity^54^ suggests that, because of waning antibodies, our serological surveillance may also underestimate population immunity to COVID-19. Second, we may overestimate the degree to which humoral immunity has waned if the assays we employ are imperfectly sensitive^55^. Indeed, it is possible that more sensitive assays are only feasible in smaller samples that are less representative of the population^55^. Third, our estimate of antibody decline due to natural infection may be incorrect if our adjusted reported case rate does not accurately estimate the infection rate across districts. In that case, our control for infections in Eq. (1) is inadequate. The fact that a 1 percentage point (p.p.) increase in that adjusted rate is associated with a 1 p.p. higher seropositivity rate, however, suggests that the adjusted rate is a reasonable measure of infections. Fourth, we may not accurately untangle seropositivity in round 3 that is due to infection versus due to vaccination. Our estimate of seropositivity among the vaccinated and among the unvaccinated during round 3 may be biased if there is selection into vaccination status that is correlated with seropositivity.

This study demonstrates the growth of seroprevalence in a large, South Indian state through two waves of the COVID-19 pandemic. Seroprevalence estimates demonstrate that officially reported infections dramatically underestimated total infections. It illustrates that humoral immunity wanes amongst those who acquire immunity via infection or vaccination. Finally, it provides a formula to decompose changes in humoral immunity into those attributable to infection versus vaccination. Going forward, estimates of seroprevalence should be updated to account for the effect of India’s third COVID-19 wave in early 2022. Moreover, complementary work that track cellular immunity over the pandemic would be valuable because cellular immunity may outlast humoral immunity^54,56–59^.

### Supplementary Information

Supplementary Information.

## Data Availability

De-identified versions of the datasets used and/or analysed during the current study available from the corresponding author on reasonable request.
