# Author Correction: Contribution of infection and vaccination to population-level seroprevalence through two COVID waves in Tamil Nadu, India

**DOI:** 10.1038/s41598-024-55994-7

**Published:** 2024-03-01

**Authors:** T. S. Selvavinayagam, Anavarathan Somasundaram, Jerard Maria Selvam, P. Sampath, V. Vijayalakshmi, C. Ajith Brabhu Kumar, Sudharshini Subramaniam, Parthipan Kumarasamy, S. Raju, R. Avudaiselvi, V. Prakash, N. Yogananth, Gurunathan Subramanian, A. Roshini, D. N. Dhiliban, Sofia Imad, Vaidehi Tandel, Rajeswari Parasa, Stuti Sachdeva, Sabareesh Ramachandran, Anup Malani

**Affiliations:** 1 Directorate of Public Health and Preventative Medicine, Government of Tamil Nadu, Chennai, Tamil Nadu India; 2 Institute of Community Medicine, Madras Medical College, Chennai, Tamil Nadu India; 3 University of California, San Diego, CA USA; 4 Artha Global, Mumbai, Maharashtra India; 5 University of Manchester, Manchester, UK; 6 World Bank, Washington D.C., USA; 7 University of Chicago, Chicago, IL USA

Correction to: *Scientific Reports* 10.1038/s41598-023-50338-3, published online 24 January 2024

In the original version of this article, Vaidehi Tandel was incorrectly affiliated with Henley Business School, University of Reading, Reading, UK.

The correct affiliation is as follows:

University of Manchester, Manchester, UK.

The original article has been corrected.

