# Supplementary material for: Contribution of infection and vaccination to population-level seroprevalence through two COVID waves in Tamil Nadu, India
Source: Sci Rep. 2024 Jan 24;14:2091. doi: 10.1038/s41598-023-50338-3 (PMC10808562; doi:10.1038/s41598-023-50338-3)
Supplement: Supplementary file 1 — Supplementary Information. [file 41598_2023_50338_MOESM1_ESM.docx]

SUPPLEMENTAL MATERIALS

Contribution of infection and vaccination to seroprevalence

through two COVID waves in Tamil Nadu, India

Selvavinayagam T.S., Somasundaram A., Jerard Maria Selvam,

Sabareesh Ramachandran, Sampath P., Vijayalakshmi V., Ajith Brabhu Kumar C.,

Sudharshini Subramaniam, Raju. S, Avudaiselvi. R, Prakash V., Yogananth N.,

Gurunathan Subramanian, Roshini. A, Dhiliban D.N., Sofia Imad, Vaidehi Tandel,

Rajeswari Parasa, Stuti Sachdeva, Anup Malani^[[1]](#footnote-1)^*

# Methods

**Sample**. Suspected or confirmed current or prior COVID-19 infection was not an exclusion criterion. If a participant was currently receiving medical care for COVID-19, a family member or proxy was used to complete the questionnaire on the participant’s behalf; however, the blood sample was taken from the participant.

**Sampling strategy**. This section provides more detail on the five steps in the study sampling strategy. *First*, districts were divided into rural and urban strata. All human settlements labeled villages in the 2011 Indian Census made up the rural strata. In rounds 1 to 3, the remaining settlements were the urban strata. (In round 4, the urban strata was further stratified into substrata comprised of municipal wards to make sure sampling was more geographically representative.) *Second*, rural and urban strata and substrata were divided into so-called clusters. In rural areas, each village was a single cluster. In urban areas, a street segment including between 50-500 households was called a cluster. *Third*, district-wise individual sample-size targets were converted into district-wise cluster sample-size targets assuming that 30 persons would be sampled per cluster. Clusters sample targets were assigned to rural and urban strata and substrata in proportion to the population of those strata. *Fourth*, simple random sampling was used to select the actual clusters to be sampled in accordance with cluster sample-size targets for each rural and urban strata or substrata.

*Finally*, within each cluster, a random GPS starting point was selected. One participant per household was sampled from households adjacent to that starting point until 30 persons consented within a cluster. Within each household, the participant asked to provide a biosample was selected via the Kish method ([1](#_ENREF_1)). If a participant refused, the survey went to next adjacent house until either 30 participants consented in the cluster or there were no more households in the cluster, whichever came first. The study asked participants using this process separately in each of the three rounds of survey; therefore, the participants sampled in each round may not be the same people sampled in other rounds.

**Data collection**. Here we provide greater detail on data collection and biospecimen analysis. Blood was collected in EDTA vacutainers. Serum was isolated and stored in Eppendorf tubes. Serum was analyzed using either of two chemiluminescent immunoassay (CLIA) kits.

The first kit was the iFlash-SARS-CoV-2 IgG kit from Shenzhen YHLO Biotech. Per the manufacturer, it has a sensitivity of 95.9% (95% CI: 93.3-97.5%) and specificity of 95.7% (95% CI: 92.5-97.6%) ([2](#_ENREF_2)). Independent analysis estimated a sensitivity of 93% (95% CI: 84.3–97.7%) and specificity of 92.9% (95% CI: 85.3–97.4%) ([3](#_ENREF_3)).

The second kit was the Vitros anti-SARS-CoV-2 IgG CLIA from Ortho-Clinical Diagnostics. Per the manufacturer it has 90% sensitivity (95% CI: 76.3-97.2%) and 100% specificity (95% CI: 99.1–100.0%) ([4](#_ENREF_4)). FDA evaluation suggests it has 100% sensitivity (95% CI: 88.7-100%) and 100% specificity (95% CI: 95.4-100%) ([5](#_ENREF_5)). Independent analysis estimated that it has a sensitivity of 98.8% (95% CI: 92.9-100%) and specificity of 97.3% (95% CI: 85-100%) ([6](#_ENREF_6)).

All the samples in a district are analyzed using the same kit in a round, with the exception of Chennai in round 1 and Virudhunagar in round 3, where different HUDs used different kits. Table S2 reports the test kit used in each district.

**Statistical analysis.** Generally, Nagapattinam district was split into Nagapattinam and Mayiladuthurai districts in March 2020, after the state started reported data on confirmed cases but before we conducted our serological survey. We aggregate these two districts together in our estimates of seropositivity and seroprevalence.

In Chennai, we do not have the population by HUDs. Since the samples were drawn proportional to population, we divide the district population across the HUDs in proportion to the sample size.

*Seroprevalence*. When estimating our district-level seroprevalence, the weights for our regression analysis employ data from the 2011 Census for the population in each age x gender category in each district. We estimate the sampling probability for demographic group (age category x sex) as the number of observations in that group in the sample in a district divided by the census population in that group in a district.

When estimating our urban- and rural-level seroprevalence, the weights for our regression analysis employ data from the 2011 Census for the population in each urban/rural category in each district. We estimate the sampling probability for urban/rural group as the number of observations in that group in the sample in a district divided by the census population in that group in a district.

We calculate the sampling probabilities for each regression observation at the level of 2011-defined districts (of which there are 32) rather than the 2020/21-defined districts (of which there are 37 or 38 depending on round), HUDs or clusters because the population is available only at the level of the old 32 districts. Likewise, we calculate district weights when we aggregate estimates across districts using the thirty-two 2001 districts. The 37 or 38, 2020/21 districts are all the same or bifurcations of the 2011 districts. Fortunately, in all bifurcated districts, the same kit was used. Therefore, we can combine all bifurcated districts into older 2011 districts for purposes of calculating sampling probabilities in regression analyses or weights when aggregating estimates.

Calculation of seroprevalence by district was modified in Chennai in round 1 and Virudhunagar in round 3. All samples in a district were tested using the same type of CLIA kit, except in Chennai and Virudhunagar, where all samples in a HUD were tested with the same type of kit. *The first step* was to calculate the weighted proportion of positive tests at the level of a health unit district (HUD), an administrative subset of districts. We estimate a weighted logit regression of test results on HUD indicators in Chennai and Virudhunagar and take the inverse logit of the coefficient for each jurisdiction indicator. Observations are weighted by the inverse of sampling probability for their age and gender groups. Clustered standard errors are calculated at the cluster level. *The second step*, for each jurisdiction, entailed predicting seroprevalence using the Rogan-Gladen formula ([7](#_ENREF_7)), test parameters for the kit used in each jurisdiction, and regression estimates of seropositive proportion by jurisdiction. In Chennai and Virudhunagar districts, we calculate seroprevalence at the district level as a weighted average of seroprevalence at the HUD level, using as weights the share of clusters in each HUD. We employ this approach to Chennai and Virudhunagar in estimators that use district-level seroprevalence.

*Attribution of changes in seropositivity to infection or vaccination*. Here we explain how we derive our formula for attributing changes in seropositivity to infection or vaccination. First, we decompose the seropositivity rate s_t_ in round t into the fraction p_t_ of the sample vaccinated by round t, the seropositivity rate s^v^_t_ among those vaccinated by round t, and the seropositivity rate s^nv^_t_ among those not vaccinated by round t:

$$s_{t}=s_{t}^{v}p_{t}+s_{t}^{nv}\left( 1-p_{t} \right).$$

The difference between the seropositivity rate in round t and t – 1 is

$$\Delta s_{t}=s_{t}-s_{t-1}=\left[ s_{t}^{v}-s_{t-1}^{v} \right]p_{t-1}+\left( s_{t}^{v}-s_{t}^{nv} \right)\left[ p_{t}-p_{t-1} \right]+\left[ s_{t}^{nv}-s_{t-1}^{nv} \right]\left( 1-p_{t-1} \right).$$

Dividing each side by the change in seropositivity across arms yields the fraction of changes in seropositivity attributable to changes in the seropositivity rate give vaccination, changes in the seropositivity rate given no vaccination, and, critically, the increase in the vaccination rate:

$$1=\frac{(s_{t}^{v}-s_{t-1}^{v})p_{t-1}}{\Delta s_{t}}+\frac{(s_{t}^{nv}-s_{t-1}^{nv})\left( 1-p_{t-1} \right)}{\Delta s_{t}}+\frac{(s_{t}^{v}-s_{t}^{nv})(p_{t}-p_{t-1})}{\Delta s_{t}}.$$

# Supplementary figures

**Figure S1. Seroprevalence by district.**


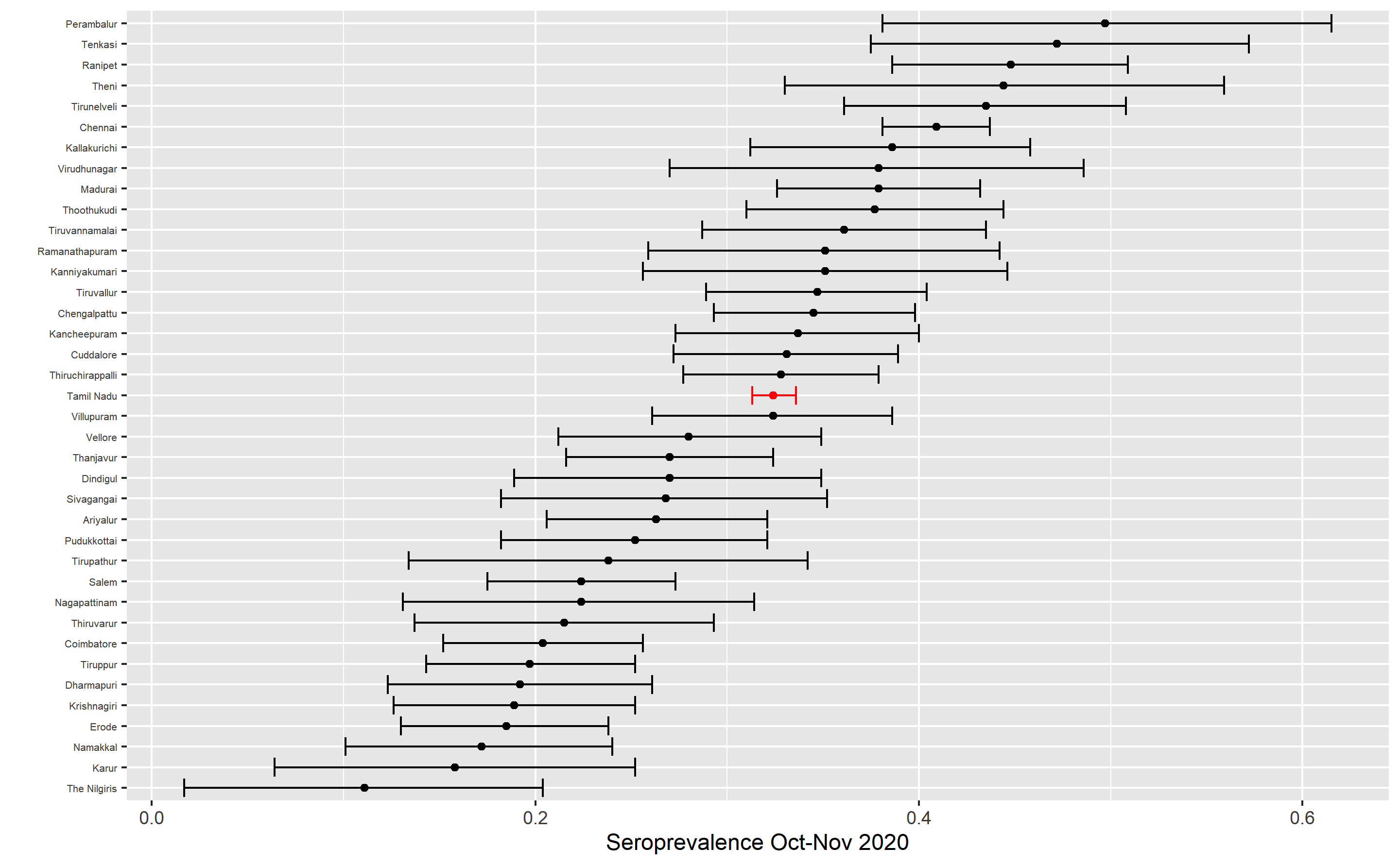

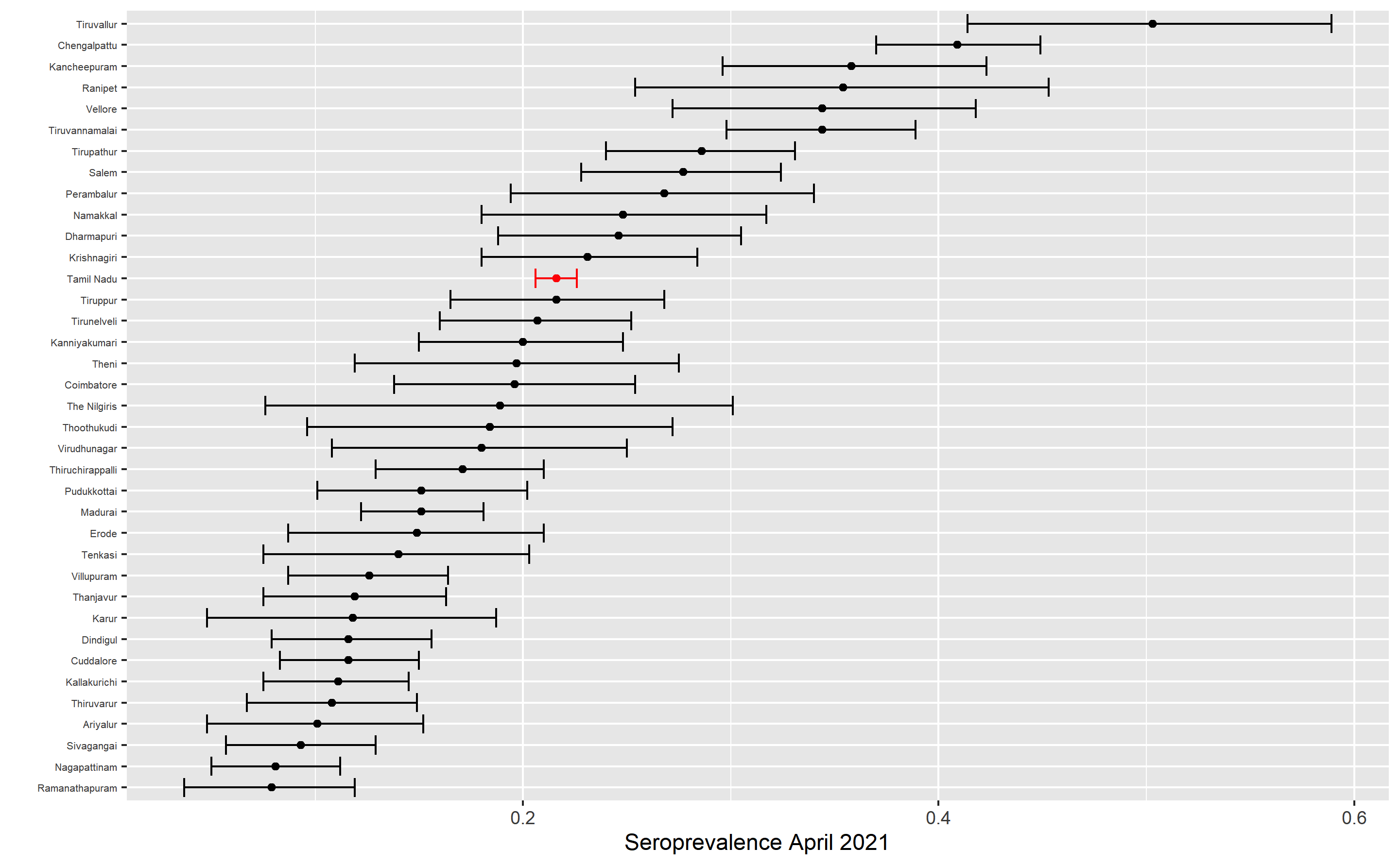


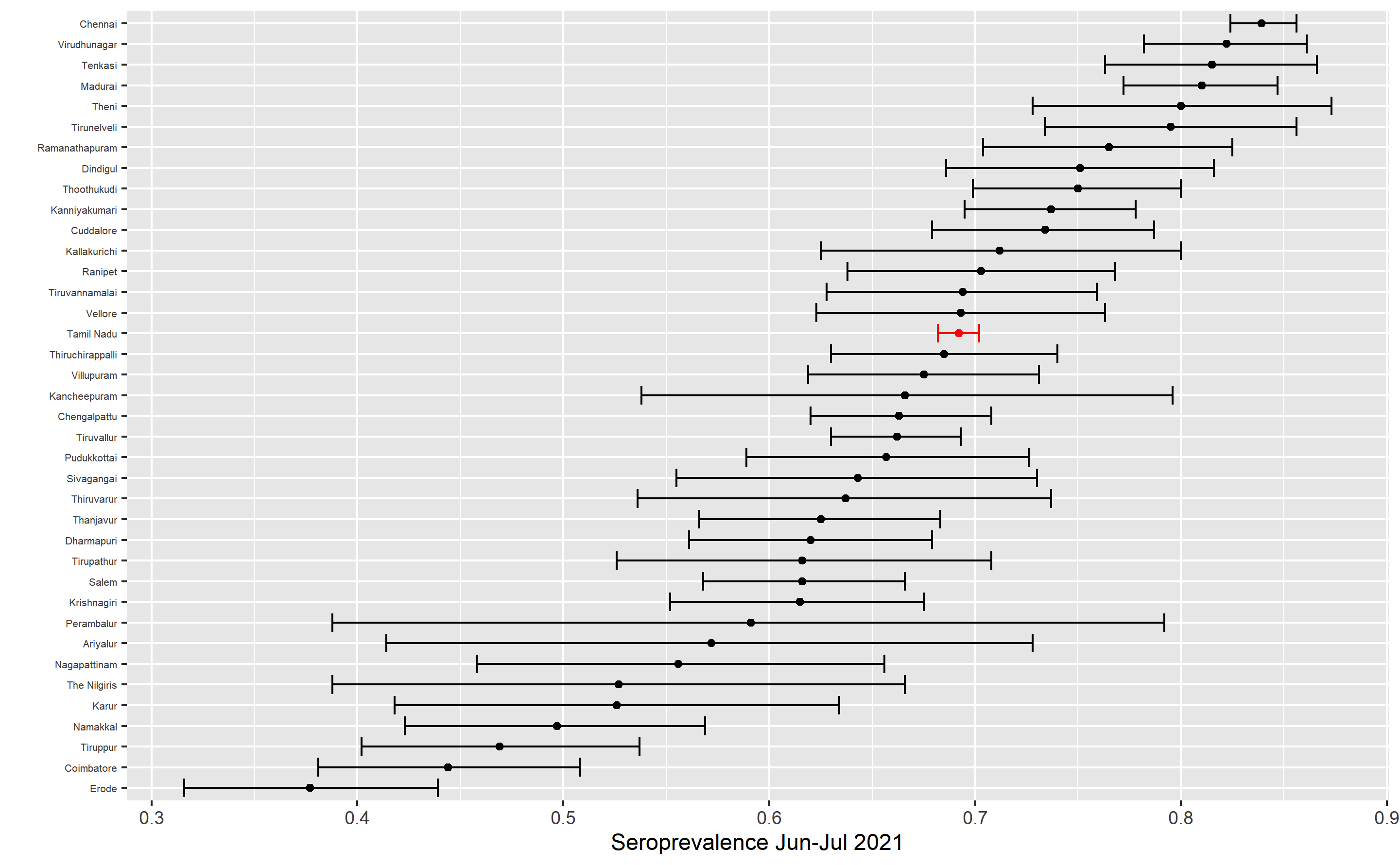

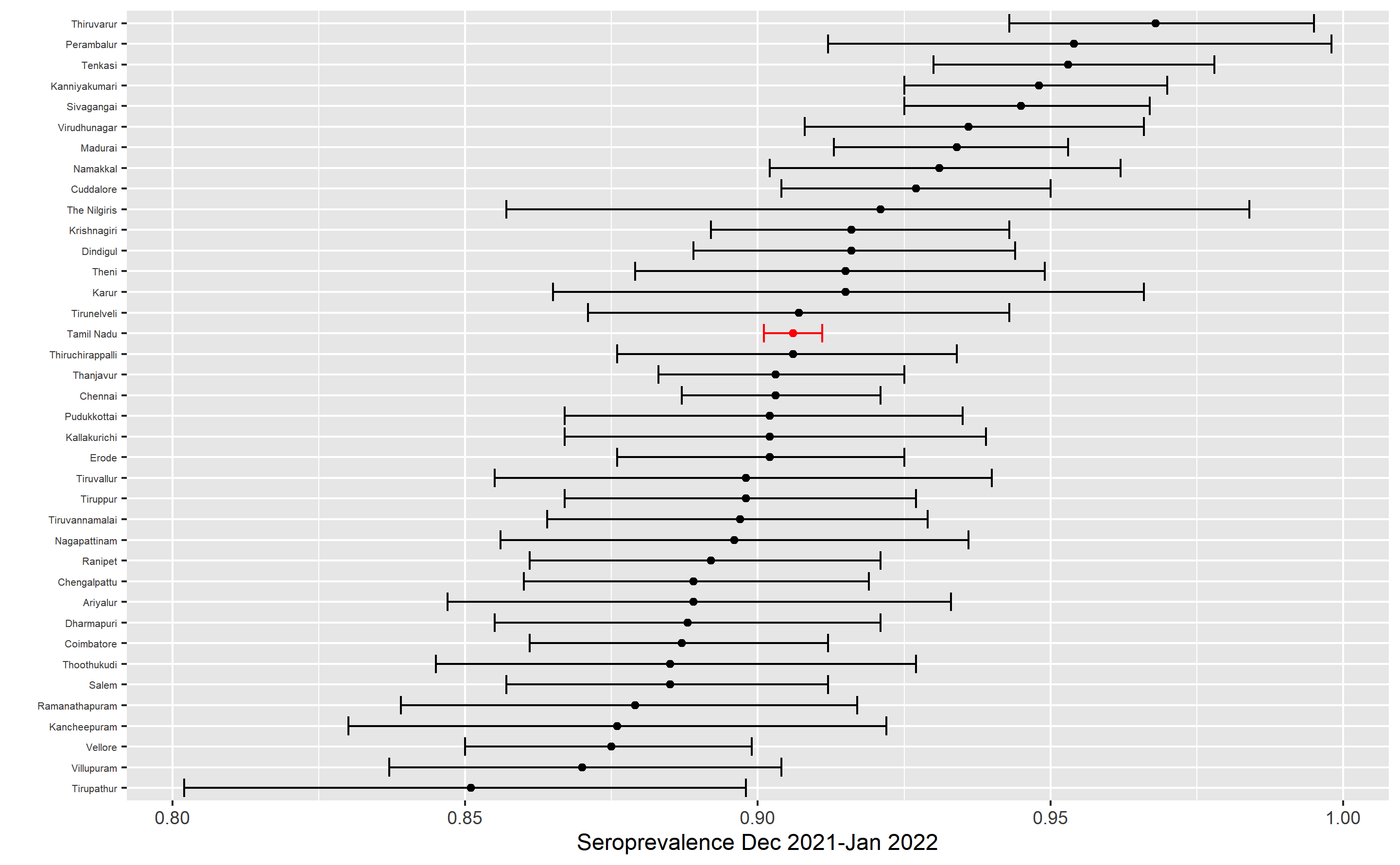


Notes: Each subfigure plots the mean (dot) and 95% confidence intervals (whiskers) of seroprevalence (based on a CLIA test for antibodies to the SARS-CoV-2 spike protein, adjusted for test accuracy using the Rogan-Gladden formula) in each district (black) and in the state overall (red) in one round of the Tamil Nadu seroprevalence survey. Districts or state are indicated on the y-axis. Weighted proportion of sample is indicated on the x-axis, with weights indicated to make the sample representative of a district or state’s population, as appropriate. The date of each round is indicated in the subfigure titles.

**Figure S2. Relationship between rate of undercounting and testing rate in Round 1.**

**
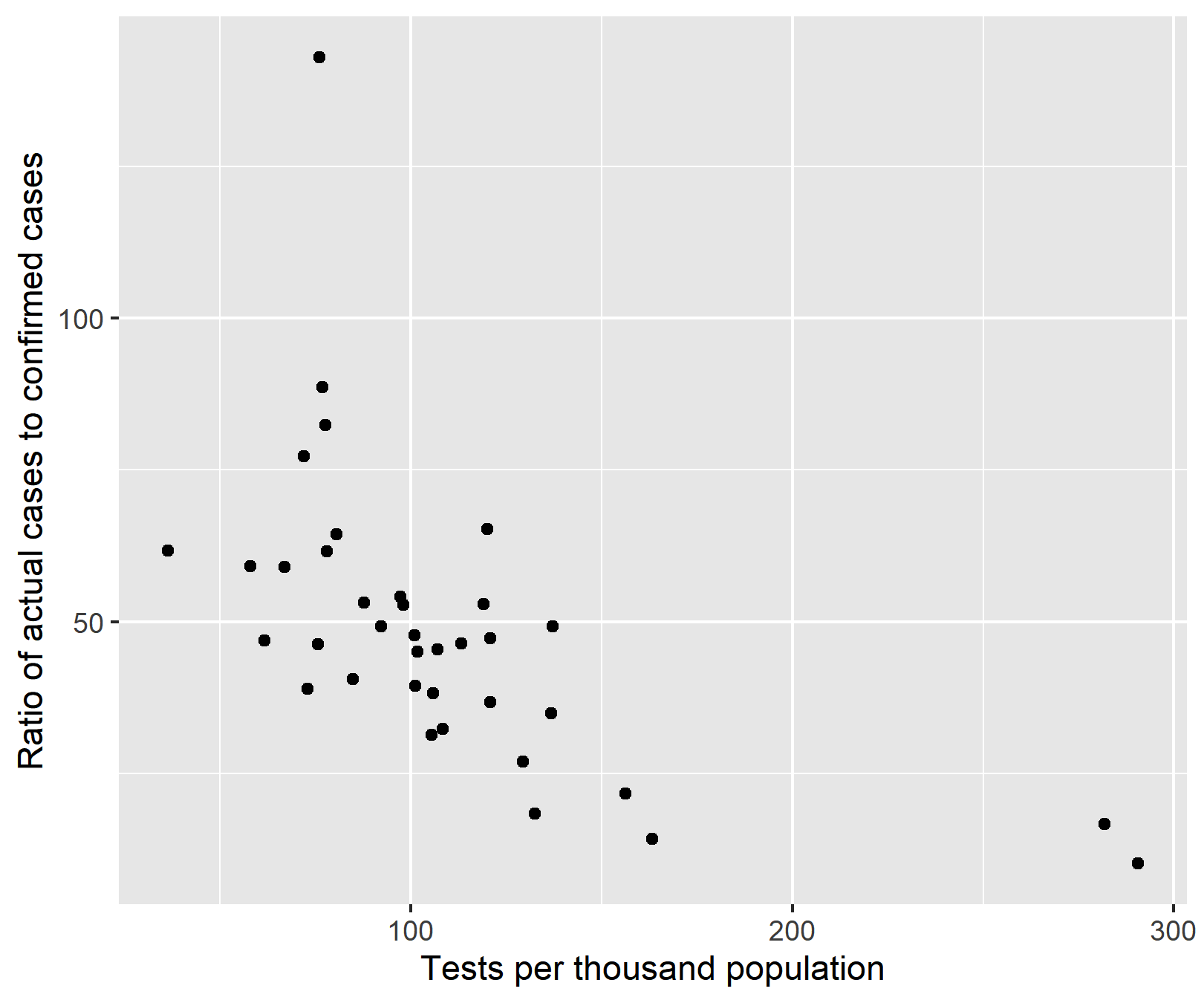
**

Notes: Figure plots the ratio of ratio of district-level undercounting of cases (from column 3 of Table 3) against the number of tests conducted per 1000 population in the district by the start of round 1 of the Tamil Nadu sero-survey. Each dot represents 1 district.

# Supplementary tables

Table S1. Confirmed cases and undercount of infections by district in round 1 (Oct. – Nov. 2020).

| District | **Sero-preva-lence** | **Total cases till survey** | **Under**  **Count ratio** |
| --- | --- | --- | --- |
| Ariyalur | 0.263 | 4191 | 50 |
| Chengalpattu | 0.345 | 40241 | 23 |
| Chennai | 0.409 | 207390 | 10 |
| Coimbatore | 0.204 | 37932 | 20 |
| Cuddalore | 0.331 | 22170 | 41 |
| Dharmapuri | 0.192 | 4954 | 62 |
| Dindigul | 0.27 | 9465 | 65 |
| Erode | 0.185 | 8644 | 51 |
| Kallakurichi | 0.386 | 9802 | 57 |
| Kancheepuram | 0.337 | 23941 | 17 |
| Kanniyakumari | 0.351 | 14158 | 49 |
| Karur | 0.158 | 3664 | 49 |
| Krishnagiri | 0.189 | 5857 | 64 |
| Madurai | 0.379 | 18014 | 68 |
| Nagapattinam | 0.224 | 5959 | 64 |
| Namakkal | 0.172 | 7845 | 40 |
| Perambalur | 0.497 | 2010 | 148 |
| Pudukkottai | 0.252 | 10001 | 43 |
| Ramanathapuram | 0.351 | 5778 | 87 |
| Ranipet | 0.448 | 14326 | 40 |
| Salem | 0.224 | 25144 | 33 |
| Sivagangai | 0.268 | 5580 | 68 |
| Tenkasi | 0.472 | 7702 | 91 |
| Thanjavur | 0.27 | 14486 | 47 |
| The Nilgiris | 0.111 | 5510 | 16 |
| Theni | 0.444 | 15888 | 37 |
| Thiruchirappalli | 0.328 | 11645 | 81 |
| Thiruvarur | 0.215 | 8620 | 33 |
| Thoothukudi | 0.377 | 14391 | 49 |
| Tirunelveli | 0.435 | 13684 | 56 |
| Tirupathur | 0.238 | 5843 | 48 |
| Tiruppur | 0.197 | 10209 | 51 |
| Tiruvallur | 0.347 | 35320 | 39 |
| Tiruvannamalai | 0.361 | 16804 | 56 |
| Vellore | 0.28 | 16854 | 28 |
| Villupuram | 0.324 | 12712 | 56 |
| Virudhunagar | 0.379 | 14980 | 52 |
| Notes: Column 3 presents the ratio of (a) the number of cases implied by seroprevalence (seroprevalence rate in column 1)in each district to (b) the number of cases reported in that district up until the date of the round 1 sero-survey in October-November 2020 (column 2). Districts are indicated in the row titles. | | | |

Table S2. Test kit used in each district.

| **District** | **Type of Kit** |  | **District** | **Type of Kit** |
| --- | --- | --- | --- | --- |
| Ariyalur | CPC Kit |  | Ramanathapuram | CPC Kit |
| Chengalpattu | Ortho Kit |  | Ranipet | Ortho Kit |
| Chennai | CPC & Ortho kits* |  | Salem | Ortho Kit |
| Coimbatore | Ortho Kit |  | Sivagangai | CPC Kit |
| Cuddalore | CPC Kit |  | Tenkasi | CPC Kit |
| Dharmapuri | Ortho Kit |  | Thanjavur | CPC Kit |
| Dindigul | CPC Kit |  | The Nilgiris | Ortho Kit |
| Erode | Ortho Kit |  | Theni | CPC Kit |
| Kallakurichi | CPC Kit |  | Thiruchirappalli | CPC Kit |
| Kancheepuram | Ortho Kit |  | Thiruvarur | CPC Kit |
| Kanniyakumari | CPC Kit |  | Thoothukudi | CPC Kit |
| Karur | CPC Kit |  | Tirunelveli | CPC Kit |
| Krishnagiri | Ortho Kit |  | Tirupathur | Ortho Kit |
| Madurai | CPC Kit |  | Tiruppur | Ortho Kit |
| Mayiladuthurai | CPC Kit |  | Tiruvallur | Ortho Kit |
| Nagapattinam | CPC Kit |  | Tiruvannamalai | Ortho Kit |
| Namakkal | Ortho Kit |  | Vellore | CPC & Ortho Kits* |
| Perambalur | CPC Kit |  | Villupuram | CPC Kit |
| Pudukkottai | CPC Kit |  | Virudhunagar | CPC & Ortho Kits* |
| Notes. 33 out of 122 clusters in Chennai used the CPC test kit in round 1. In round 3, all clusters in Chennai used the Ortho kit. Virudhunagar used only the CPC kit in rounds 1 and 2. In round 3, in 15 out of 23 clusters in Virudhunagar CPC kits were used. In Vellore Ortho kits were used in rounds 1 and 2 and Ortho kits in round 3. In round 4 Ortho kits were used in all districts. | | | | |

# References

1. Kish L. A Procedure for Objective Respondent Selection within the Household. Journal of the American Statistical Association. 1949;44(247):380-7.

2. Shenzhen YHLO Biotech Co. Ltd. Customer Notification: Sensitivity and Specificity of iFlash-SARS-Cov-2 IgG and IgM kits from Clinical Trials 2020.

3. Plebani M, Padoan A, Negrini D, Carpinteri B, Sciacovelli L. Diagnostic performances and thresholds: The key to harmonization in serological SARS-CoV-2 assays? Clinica Chimica Acta. 2020;509:1-7.

4. Ortho Clinical Diagnostics. INSTRUCTIONS FOR USE - CoV2G (Version 4.2). 2020. Contract No.: Pub. No. GEM1292_US_EN.

5. U.S. Food and Drug Administration. Serology Test Evaluation Report for “VITROS Immunodiagnostic Products Anti-SARS-CoV-2 IgG Reagent Pack” from Ortho-Clinical Diagnostics, Inc.; 2020 October 3, 2020.

6. Theel ES, Harring J, Hilgart H, Granger D. Performance Characteristics of Four High-Throughput Immunoassays for Detection of IgG Antibodies against SARS-CoV-2. Journal of Clinical Microbiology. 2020;58(8):e01243-20.

7. Rogan WJ, Gladen B. Estimating prevalence from the results of a screening test. American journal of epidemiology. 1978;107(1):71-6.

1. * Directorate of Public Health & Preventative Medicine, Government of Tamil Nadu: Selvavinayagam, T.S., Somasundaram A., Jerard Maria Selvam, Sampath P., Vijayalakshmi V., Ajith Brabhu Kumar C., Sudharshini Subramaniam, S. Raju, Avudaiselvi, Prakash V., Yogananth N., Roshini, and Dhilipan; University of California, San Diego: Sabareesh Ramachandran; IDFC Institute: Sofia Imad. Rajeshwari Parasa; Independent: Vaidehi Tandel and Stuti Sachdeva; University of Chicago: Anup Malani. Corresponding author ([amalani@uchicago.edu](mailto:amalani@uchicago.edu)). [↑](#footnote-ref-1)
